# Inhibition of PKC‐δ reduce rhabdomyolysis‐induced acute kidney injury

**DOI:** 10.1111/jcmm.17331

**Published:** 2022-05-02

**Authors:** Dengke Wu, Jian Pan, Dongshan Zhang

**Affiliations:** ^1^ Department of Emergency Medicine Second Xiangya Hospital of Central South University Changsha China; ^2^ 70566 Emergency Medicine and Difficult Diseases Institute Second Xiangya Hospital Changsha China

**Keywords:** AKI, apoptosis, ERK1/2, p38, PKC‐δ

## Abstract

Despite extensive research, the mechanisms underlying rhabdomyolysis‐induced acute kidney injury (AKI) remain largely elusive. In this study, we established both cell and murine models of rhabdomyolysis‐induced AKI by using myoglobin and glycerin, respectively, and provided evidence that protein kinase Cδ (PKC‐δ) was activated in both models and subsequently promoted cell apoptosis. Moreover, we found that this detrimental effect of PKC‐δ activation can be reversed by its pharmaceutical inhibitor rottlerin. Furthermore, we detected and confirmed the existence of PKC‐δ‐mediated myoglobin‐induced cell apoptosis and the expression of TNF‐α and IL1‐β *via* regulation of the p38MAPK and ERK1/2 signalling pathways. In summary, our research revealed the role of PKC‐δ in renal cell apoptosis and suggests that PKC‐δ is a viable therapeutic target for rhabdomyolysis‐induced AKI.

## 1 INTRODUCTION

Rhabdomyolysis is a syndrome caused by the breakdown of skeletal muscle and necrosis resulting from various conditions. The release of intracellular contents and breakdown products into the bloodstream can lead to acute kidney injury (AKI), a life‐threatening complication associated with poor outcomes including end‐stage renal disease (ESRD) and even death.^1^, ^2^, ^3^ Trauma patients often endure direct muscle injury; damaged myocytes can release myoglobin, creatine phosphokinase, and lactate dehydrogenase into the circulation, thus creating a heavy burden on the kidneys.^4^, ^5^, ^6^ At present, the mechanisms underlying rhabdomyolysis‐related AKI are not well understood despite significant effort, leaving us with limited options for the prevention and treatment of this condition. Tubular damage, and more specifically apoptosis of the tubule cells, is the main pathological change occurring during AKI. Apoptosis is the main form of programmed cell death and renal cell apoptosis is a necessary aspect of normal renal function.^7^, ^8^ Under normal circumstances, apoptotic cells turn into small apoptotic bodies that are either ingested by phagocytes or taken up by the epithelia into the tubular lumen.^9^, ^10^ However, apoptosis causes the loss of parenchymal cells during the process of acute renal injury. This is because there are more apoptotic cells than neighbouring cells; these cells progress into a necrosis phase, spilling toxic and immunogenic contents that cause further damage in the kidney.^10^ Exploring the mechanisms underlying renal cell apoptosis might provide us with an interventional method for AKI at an earlier stage.

Protein kinase C is a subgroup of serine/threonine kinases that when activated regulate multiple cellular functions, including cell differentiation, survival and death.^11^, ^12^, ^13^ Phosphorylated protein kinase C has been reported to influence several renal diseases in manners that both expedite or impede apoptosis; furthermore, isoform‐specific inhibitors and agonists have been proven to counter or promote these effects.^14^, ^15^ PKC‐δ, one of the key PKC isoforms, is known to play an anti‐apoptosis role in tumour cells.^16^ However, recent studies showed that PKC‐δ promotes renal cell apoptosis under treatments with cisplatin and vancomycin, and in cold storage transplantation‐induced AKI.^17^, ^18^, ^19^ At present, the specific role and mechanisms underlying PKC‐δ in rhabdomyolysis‐induced AKI remain unclear.

In the current study, we found that PKC‐δ was induced by myoglobin and glycerin both in vitro and in vivo. We also found that the inhibition of PKC‐δ by both rottlerin and PKC‐δ‐KD attenuated the progression of rhabdomyolysis‐induced AKI and improved the survival rate. Mechanistically, we used mouse proximal tubular cells to demonstrate that the inhibition of PKC‐δ ameliorated myoglobin‐induced renal cell apoptosis *via* the inactivation of the p38MAPK and ERK1/2 pathways. In summary, our data suggested that the inhibition of PKC‐δ ameliorates rhabdomyolysis‐induced apoptosis in renal epithelial cells and kidney injury.

## 2 METHOD AND MATERIALS

### 2.1 Reagents and antibodies

Antibodies were obtained from the different sources. PKC‐δ (2058), phospho‐PKC‐δ (9374), Caspase3 (9662), cleaved caspase3 (9664), p38 (8690), phospho‐p38 (4511) and phospho‐p44/42 MAPK (Erk1/2) (4370) were from Cell Signaling Technology (Danvers, MA, USA); anti‐Erk1/2 (ab184699) from Abcam (Cambridge, UK); β‐Tubulin (T0023) were from affinity (UK); Myoglobin and ascorbic acid were from Sigma‐Aldrich (St. Louis, MO, USA). Rottlerin (the inhibitor of PKC) were from Abcam (Cambridge, UK);

### 2.2 Animal model of RM‐induced AKI

C57BL/6 mice were purchased from Hunan SJA Laboratory Animal Co., Ltd (Changsha, China). Male C57BL/6 mice aged 8–10 weeks were injected 50% glycerol at a dose of 8ml/kg as previous described^20^; the control group was injected with an equal volume of saline. The mice were injected intraperitoneally with Rottlerin were 10 mg/kg/day to suppress the PKC. Animal experimental protocols were approved by the Care and Use of Laboratory Animals Institutional Committee from Second Xiangya Hospital, China. The mice were housed at stable room temperature in a 12‐h light/dark cycle and provided adequate supplies of standard rodent chow and water. We randomly divide twenty mice aged 6–8 weeks into two groups, and mice were intraperitoneally injected with saline or glycerol. We observe the survival of mice within 48 h to obtain the survival curve of mice after injection of glycerol.

### 2.3 Cell culture and treatment

We obtained the BUMPT cell line (mouse renal proximal tubular epithelial cell) from Dr. Wilfred Lieberthal of Boston University School of Medicine. BUMPT cells were cultured with Dulbecco's modified Eagle's medium (Gibco) added with 10% foetal bovine serum (Gibco) and 1% penicillin–streptomycin in 5% CO2 at 37℃. Myoglobin and ascorbic acid were added into the medium for 24 h to establish the RM‐induced AKI in vitro as described previously.^20^, ^21^ Cells were plated in 35‐mm dish to reach 50%–70% density and then transfected with 1 μg PKC plasmids (PKC‐δ‐KD, PKC‐δ‐CF) using Lipofectamin 2000 (Invitrogen) or as previously described. After 6 h of transfection, cells were treated with myoglobin and ascorbic acid. For p38 or ERK1/2 inhibition, BUMPT cells were treated with saline or myoglobin (10mg/ml) for 24 h, with or without p38 MAPK inhibitor(SB203580,5 μM) or ERK1/2 inhibitor(SCH772984,300 nM) preincubation for 1 h.

### 2.4 Real‐time PCR

We use TRIzol (Takara, T9108) to extract RNA from tissues and cells according to the instructions and use a reverse transcription kit (Takara, RR037A) to prepare cDNA as previously described, and then, use SYBR green kit (AG,11701) to detect the following different indicators: TNF‐α: forward 5′‐TAGCCAGGAGGGAGAACAGA‐3′, reverse 5′‐TTTTCTGGAGGGAGATGTGG‐3′; IL‐1β: forward 5′‐CCCAAGCAA TACCCAAAGAA‐3′, reverse 5′‐GCTTGTGCTCTGCTTGTGAG‐3′; GADPH: forward 5′‐TG CTGAGTATGTCGTGGAGTCTA‐3′, reverse 5′‐AGTGGGAGTTGCTGTTG AAATC‐3′.

### 2.5 Western blot

We use RIPA Lysis Buffer (Beyotime, P0013C) to extract protein from tissues and cells according to the protocol and perform experiments by SDS‐PAGE, then transfer the protein to the PVDF membrane. Use 5% BSA to block the membrane. After this, incubate the membrane with primary antibody overnight (PKC‐δ 1:1000, p‐PKC‐δ 1:1000, Caspase3 1:1000, cleaved caspase3 1:1000, p38 1:1000, p‐p38 1:1000, anti‐Erk1/2 1:1000, p ‐Erk1/2 1:1000, β‐Tubulin 1:2000). After washing the membrane with PBS three times, incubate the membrane with second antibody 1 h at room temperature. Finally, we observe the protein band under the instrument (Tanon) through the ECL reagent.

### 2.6 TUNEL and HE staining

Tissue is embedded in paraffin and sliced. The sections are deparaffinized and stained differently according to the TUNEL kit (Roche, 11684817910) and HE staining kit as previously described.^18^, ^22^, ^23^, ^24^, ^25^, ^26^ Histologic changes in the cortex and the outer stripe of the outer medulla (OSOM) were scored by the percentage of renal tubules with loss of brush border, cellular necrosis, cast formation, tubule dilation and vacuolization (0, no damage; 1, <25%; 2, 25%–50%; 3, 50%–75%; 4, >75%). For the analysis of TUNEL,^17^, ^27^ we randomly selected 10–20 fields from each tissue section to count the TUNEL‐positive cells per millimetre.

### 2.7 Statistics

All data were presented as means ± SD. Two‐tailed Student t tests were used for two group comparisons. Two‐way ANOVA was used for the multiple group comparisons. *p* < *0*.*05* was considered statistically significant.

## 3 RESULTS

### 3.1 PKC‐δ was activated by glycerine‐induced AKI

**FIGURE 1:**
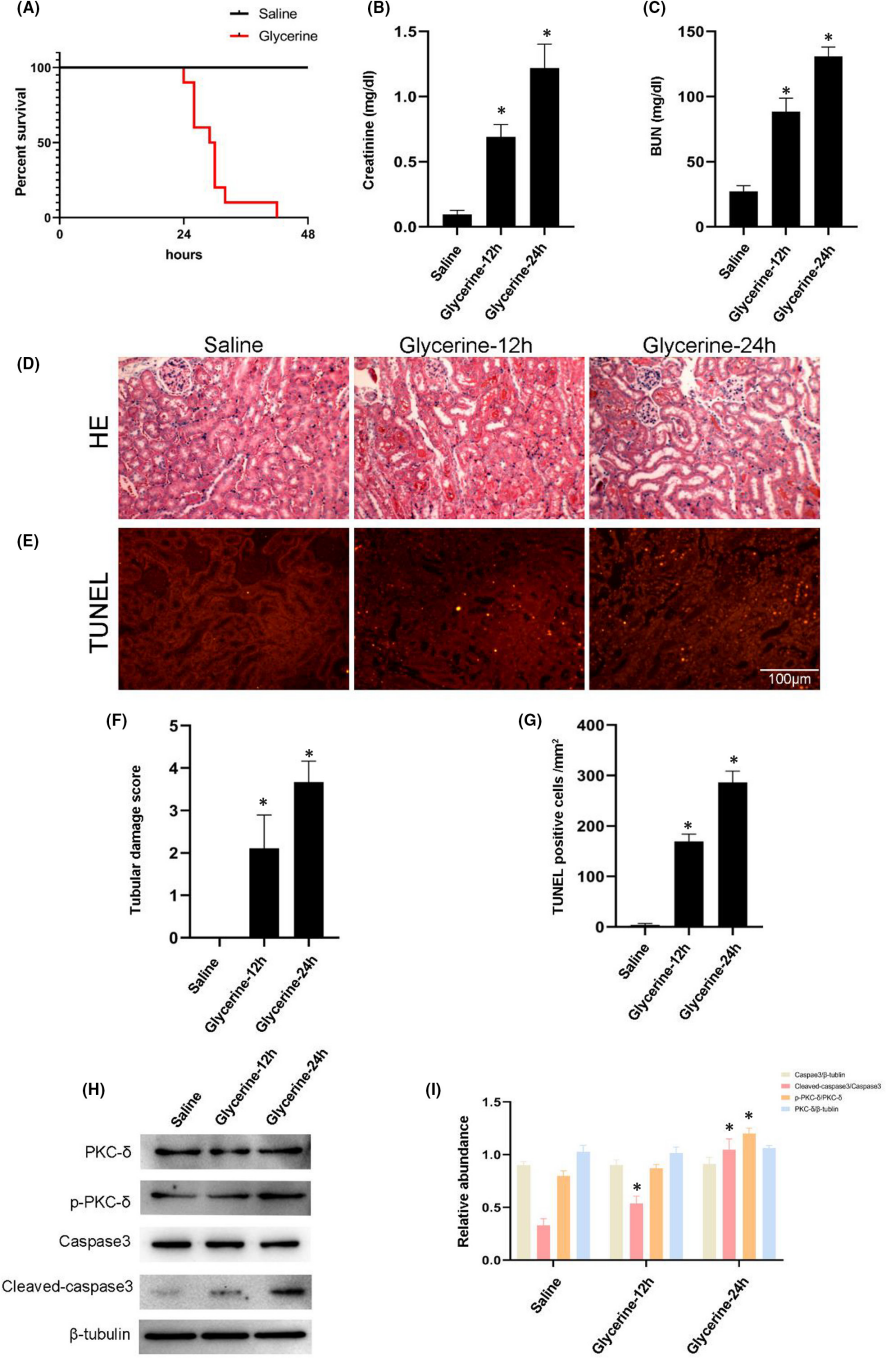
Glycerine induced renal injury in a time‐dependent manner. WT mice were injected intramuscularly with saline or 50% glycerine (8 ml/kg) for 12 or 24 h. (A) Survival rate of WT mice after saline or glycerine treatment for 0h, 24, 36 or 48 h. (B,C) Serum creatinine and BUN measurement. (D,E) Representative images of H&E staining. (F,G) Quantitative analysis of tubular damage. (H) Representative immunoblots of PKC‐δphosphorylation and Caspase3 activation in whole kidney lysate. (I) Grayscale image analysis between them. Original magnification, ×200. Scale bar, 100 μM. Data are expressed as mean ± SD (*n* = 6). * *p* < 0.05 versus Saline group

Prior to detecting PKC‐δ, we wanted to establish and validate a murine model of rhabdomyolysis‐induced AKI. To investigate survival rates, C57BL/6 mice were intramuscularly injected with glycerin at a dose of 14 µl/g for 48 h; resultant data suggested that C57BL/6 mice began to die at 24 h and all mice had died by the 40 h timepoint (Figure 1A). To create a model of AKI, C57BL/6 mice were intramuscular injected with glycerin a dose of 14 µl/g for 12 and 24 h. Functionally, the levels of serum creatinine and blood urine were notably increased at 12 h and reached a peak at 24 h (Figure 1B,C). H&E staining showed that glycerin induced tubular damage and tube type at 12 h and induced moderate levels of damage by 24 h (Figure 1D); this was supported by the tubular damage score (Figure 1F). TUNEL staining results indicated that glycerine induced renal cell apoptosis at 12h, and that the levels of apoptosis had increased further by 24 h; this effect was demonstrated by the counting of TUNEL‐positive cells (Figure 1E,G). Also, PI staining indicated elevated cell death in a time‐dependent manner in mice kidney (Figure S1A,B). Finally, immunoblotting results indicated that glycerine induced the activation of PKC‐δ and caspase3 at 12 h; with notable increased levels at 24 h (Figure 1H,I). Further immunoblotting detection showed phosphorylated p38 and ERK1/2 expression were increased too in glycerin‐treated mice (Figure S1 C,D). We complementarily detected the expression of other PKC‐α and PKC‐η and found that they were also activated by glycerin (Figure E,F). Taken together, these data suggested that PKC‐δ was activated by glycerine in C57BL/6 mice.

### 3.2 Rottlerin attenuated glycerine‐induced AKI

**FIGURE 2:**
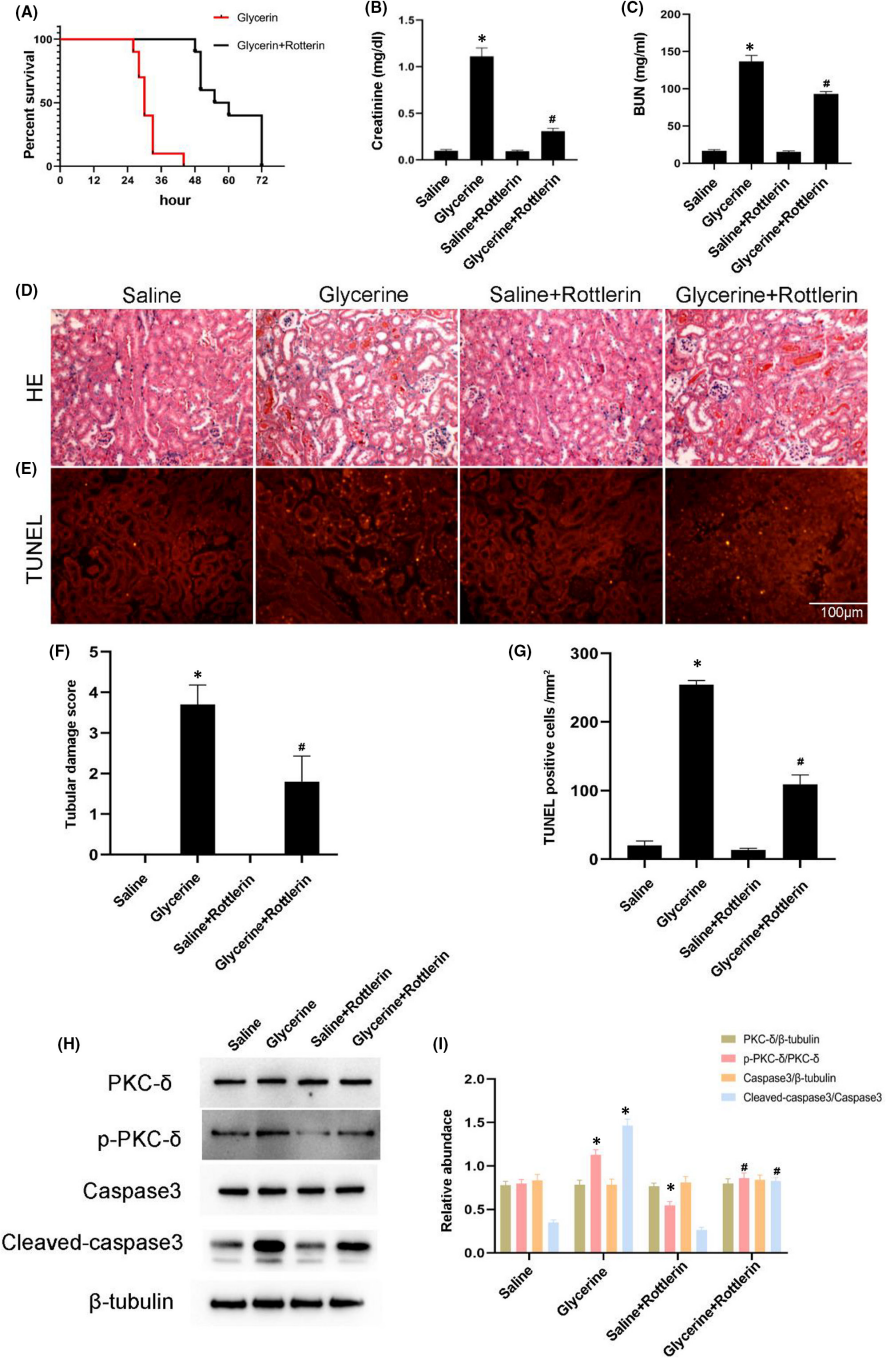
PKC‐δ inhibitor Rottlerin attenuates renal injury induce by glycerine. WT mice were injected intramuscularly with saline or 50% glycerine (8 ml/kg), with or without Rottlerin 10 mg/kg for 24 h. (A) Survival rate of WT mice after saline or glycerine injection and Rottlerin treatment for 0, 24, 36 or 48 h. (B,C) Serum creatinine and BUN measurement. (D) Representative images of H&E staining. (E) Representative images of TUNEL staining. (F). Quantitative analysis of tubular damage. (G) Counting of TUNEL‐positive cells showing Rottlerin reduced apoptosis in glycerine induced renal injury. (H) Representative immunoblots of PKC‐δ phosphorylation and Caspase3 activation in whole kidney lysate. (I) Grayscale image analysis between them. Original magnification, ×200. Scale bar, 100 μM. Data are expressed as mean ± SD (*n* = 6). * *p* < 0.05 versus Saline group. # *p* < 0.05 versus glycerine group

To investigate the role of PKC‐δ in rhabdomyolysis‐induced AKI, rottlerin, a pharmacological inhibitor, was used to suppress the activation of PKC‐δ. Firstly, rottlerin notably attenuated glycerine‐induced death in C57BL/6 mice (Figure 2A). Secondly, glycerine‐induced a decline in renal function; this was ameliorated by rottlerin treatment at 24 h (Figure 2B,C). H&E staining indicated that rottlerin also reduced glycerine‐induced renal tubular damage and tube type; this effect was supported by the tubular damage score at 24 h (Figure 2D,F). Thirdly, TUNEL staining results demonstrated that rottlerin also suppressed glycerine‐induced renal cell apoptosis at 24 h (Figure 2E,G). Immunoblot results showed that rottlerin markedly attenuated the glycerine‐induced activation of PKC‐δ and caspase3 at 24 h (Figure 2H,I). We then injected mice with rottlerin after glycerin treatment and found that rottlerin significantly inhibited the activation of phosphorylated p38 and ERK1/2 (Figure S2). In summary, these data suggested that the inhibition of PKC‐δ attenuates AKI caused by glycerine.

### 3.3 Rottlerin and PKCδ‐KD plasmid attenuated renal damage caused by Glycerin and MYO in mice

**FIGURE 3:**
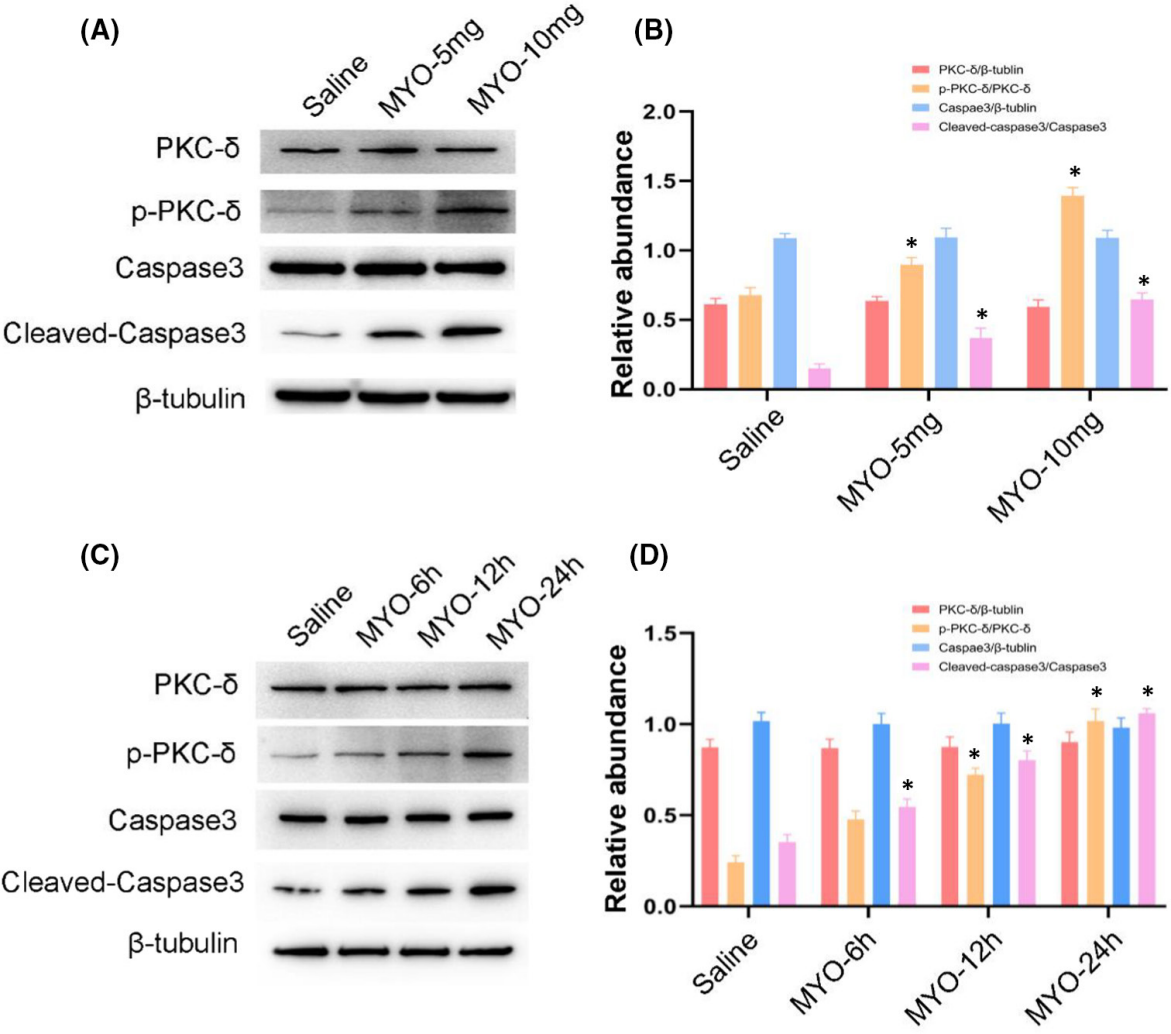
Myoglobin induces apoptosis and PKC‐δ phosphorylation in BUMPT cells. BUMPT cells were treated with myoglobin in different doses or for different durations. (A,C) Representative immunoblots of PKC‐δ phosphorylation and Caspase3 activation in cell lysate. (B,D) Grayscale image analysis between them. Data are expressed as mean ± SD (*n* = 6). * *p* < 0.05 versus Saline group

We then tried to pin the function of PKC‐δ in rhabdomyolysis induced AKI in vivo. We injected the mice with saline or rottlerin (10 mg/kg/day) for three days before they underwent intramuscular glycerin injection. 24 h later, mice kidneys were collected, H&E staining showed markedly decreased tubular damage (Figure S3A,C), and TUNEL staining detected far less apoptosis in mice treated with rottlerin (Figure S3B,D), indicating that inhibition of PKC kinase exerts a renal protective function. To specify the role of PKC‐δ, we injected mice with 25 μg PKC‐δ‐KD plasmid through tail vein per day for three day before intramuscular glycerin injection and found that the activation of PKC‐δ p38 MAPK and ERK1/2 were all down‐regulated significantly in PKC‐δ‐KD‐treated mice by immunoblotting (FigureS 3B,D). These results together confirmed that PKC‐δ inhibition can protect kidney from crush syndrome‐related renal injury,

### 3.4 PKC‐δ was activated by myoglobin in BUMPT cells

To further investigate the role of PKC‐δ, we used myoglobin in a range of *in vitro* experiments. Immunoblotting results indicated that myoglobin induced the activation of PKC‐δ and caspase3 in a dose‐dependent manner (5 and 10 mg/ml) (Figure 3A,B). In addition, myoglobin (10 mg/ml) induced the activation of PKC‐δ and caspase3 in a dose‐dependent manner (6, 12, and 24 h) (Figure 3C,D). These data showed that myoglobin induced the activation of PKC‐δ in BUMPT cells.

### 3.5 Rottlerin attenuated myoglobin‐induced apoptosis in BUMPT cells

**FIGURE 4:**
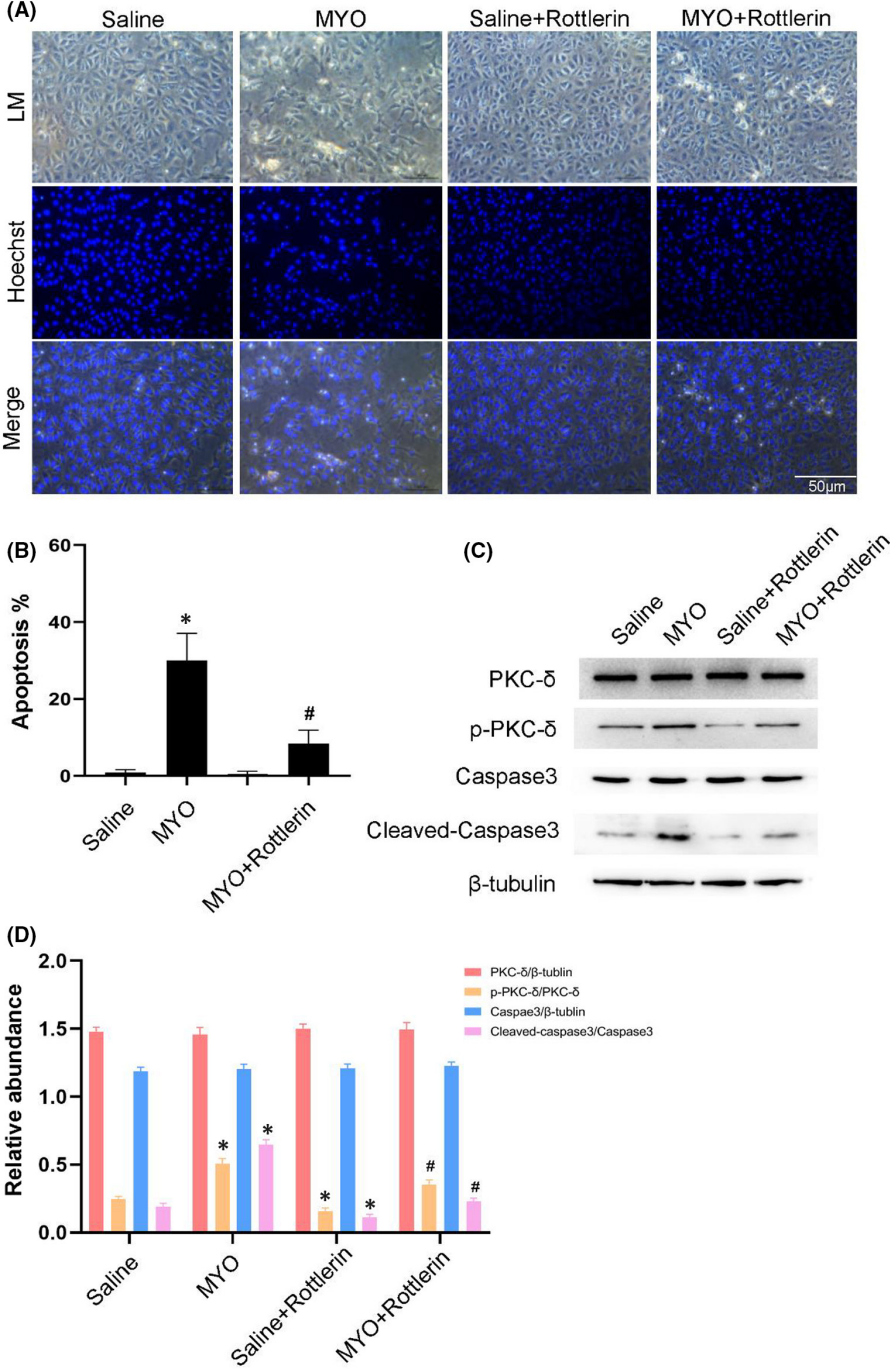
PKC‐δ inhibitor Rottlerin attenuates cell injury induced by myoglobin. BUMPT cells were treated with saline or myoglobin (10 mg/ml), with or without Rottlerin for 24 h. (A) Representative cell images combined with Hoechst staining. (B) Apoptosis rate quantified by counting of the cells with typical apoptotic morphologies. (C) Representative immunoblots of PKC‐δ phosphorylation and Caspase3 activation in cell lysate. (D) Grayscale image analysis between them. Magnification, ×400. Scale bar, 50 μM. Data are expressed as mean ± SD (*n* = 6). * *p* < 0.05 versus Saline group. # *p* < 0.05 versus MYO‐treated group

Hoechst staining demonstrated that rottlerin attenuated myoglobin‐induced apoptosis in BUMPT cells. This was confirmed by determining the rate of apoptosis (Figure 4A,B). Rottlerin notably attenuated glycerine‐induced activation of PKC‐δ and caspase3 at 24 h (Figure 4C,D). These findings were consistent with the *in vivo* results.

### 3.6 PKC‐δ‐mediated myoglobin induced BUMPT cell apoptosis and the expression of TNF‐α and IL1‐β by regulating the p38MAPK and ERK1/2 signalling pathways

**FIGURE 5:**
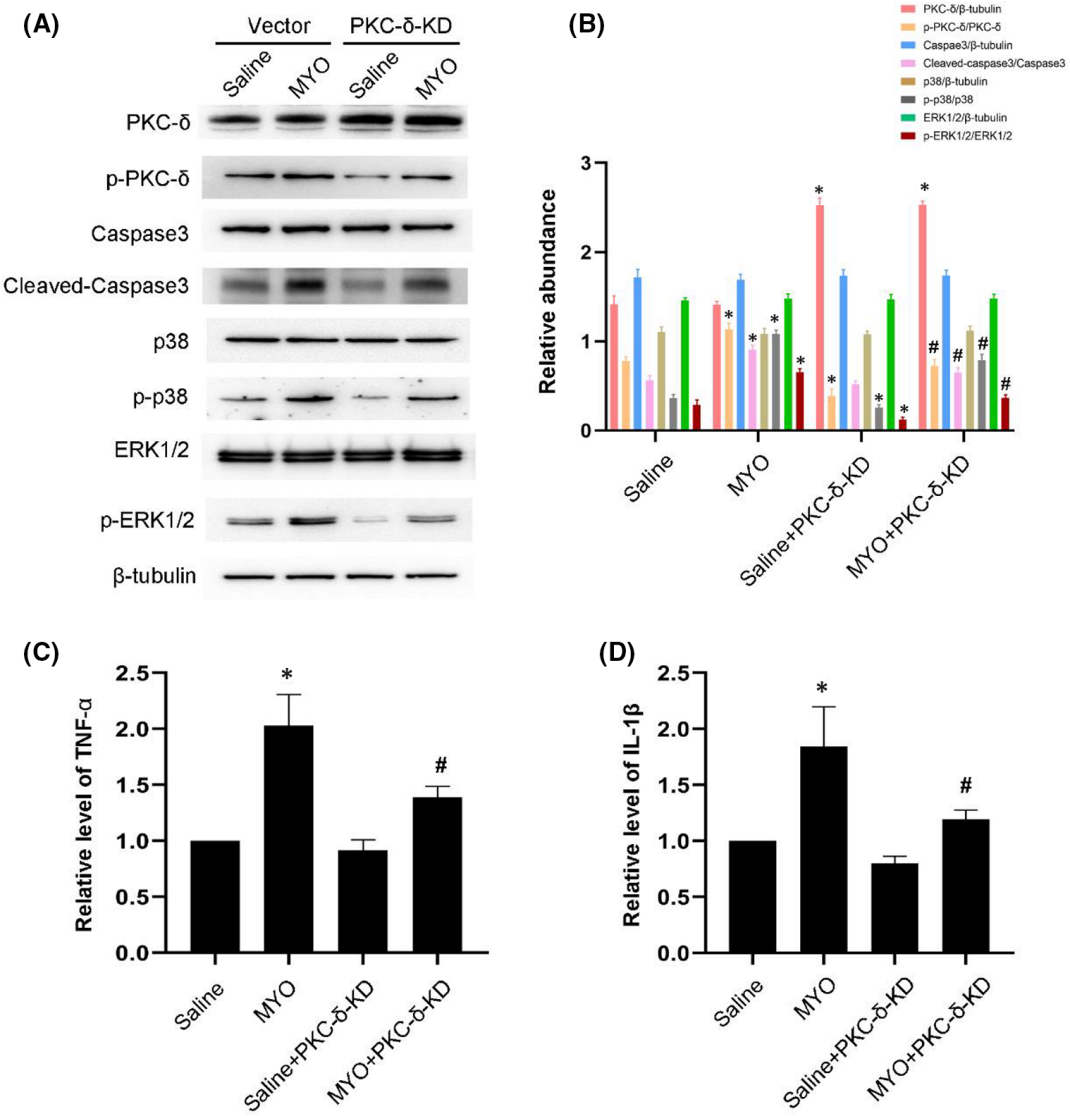
PKC‐δ‐KD attenuates cell injury induced by myoglobin. BUMPT cells were treated with saline or myoglobin (10 mg/ml), with or without transfection of 1 μg kinase dead PKC‐δ (PKC‐δ‐KD) for 24 h. (A) Representative immunoblots of PKC‐δ phosphorylation, Caspase3 activation, p38 MAPK phosphorylation and ERK1/2 phosphorylation in cell lysate. (B) Grayscale image analysis between them. (C,D) the mRNA level of TNF‐α and IL‐1β. Data are expressed as mean ± SD (*n* = 6). * *p* < 0.05 versus Saline group. #*p* < 0.05 versus MYO‐treated group

**FIGURE 6:**
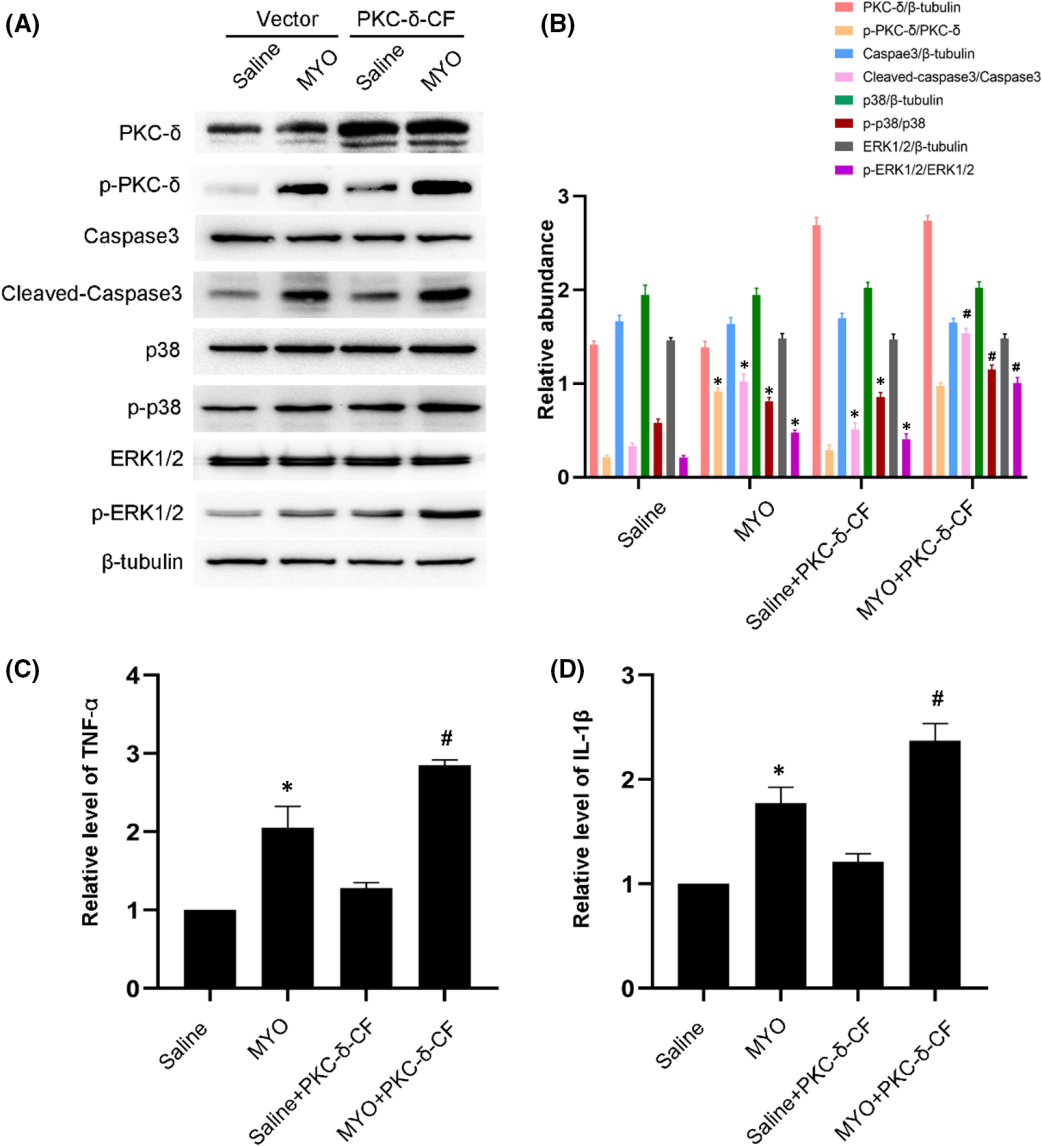
PKC‐δ‐CF aggravates cell injury induced by myoglobin. BUMPT cells were treated with saline or myoglobin (10 mg/ml), with or without transfection of 1 μg PKC‐δ active fragment (PKC‐δ‐CF) for 24 h. (A) Representative immunoblots of PKC‐δ phosphorylation, Caspase3 activation, p38 MAPK phosphorylation and ERK1/2 phosphorylation in cell lysate. (B) Grayscale image analysis between them. (C,D) the mRNA level of TNF‐α and IL‐1β. Data are expressed as mean ± SD (*n* = 6). *p < 0.05 versus Saline group. #*p* < 0.05 versus MYO‐treated group

Considering the negative effect of rottlerin, we selected PKC‐δ‐KD as a specific inhibitor of PKC‐δ. The transfection rate was detected by immunofluorescence imaging of BUMPT cells transfected with GFP + vector (Figure S4A). Besides, PKC‐δ and p‐PKC‐δ expression in BUMPT cells was down‐regulated by PKC‐KD transfection and up‐regulated by PKC‐CF transfection (Figure S4B,C). Immunoblotting results indicated that myoglobin induced the activation of PKC‐δ, caspase3, p38MAPK and ERK1/2; this effect was most notable in the PKC‐δ‐KD treatment (Figure 5A,B). In addition, RT‐qPCR analysis demonstrated that PKC‐δ‐KD markedly suppressed the myoglobin‐induced expression of TNF‐α and IL1‐β (Figure 5C,D). In contrast, these effects were significantly enhanced by the overexpression of PKC‐δ‐KD (Figure 6). These data suggested that the PKC‐δ/p38MAPK and ERK1/2 axis mediated myoglobin‐induced apoptosis in renal cells and the production of inflammatory factors.

### 3.7 P38 MAPK and ERK1/2 inhibitor attenuated MYO‐induced BUMPT cell apoptosis

Immunoblotting results indicated that BUMPT cell apoptosis induced by MYO was attenuated after preincubation with p38 or ERK1/2 inhibitor, respectively, while expression of PKC‐δ and p‐PKC‐δ remained unaffected, suggesting PKC‐δ influenced renal cell apoptosis via p38MAPK and ERK1/2 pathway(Figure S5).

## 4 DISCUSSION

Proximal tubular cell damage is closely associated with AKI. Rhabdomyolysis refers to the breakdown of striated muscle resulting from traumatic events, genetic issues, exertional and non‐exertional conditions and can cause renal damage due to the release of cellular contents from damaged muscle cells into the bloodstream; these secretions will eventually poison the kidney. The typical clinical presentation of this condition is pain and weakness in the muscle and the discoloration of urine. Another common factor that is toxic to renal cells is cisplatin. Cisplatin is a common treatment for multiple types of cancers and exerts a highly toxic effect on the kidneys, thus contributing to renal failure, a severe side effect of chemotherapy. Cisplatin‐induced AKI is reported to be facilitated by tubular cell apoptosis and necrosis in both patients and animal models.^28^, ^29^ According to Jianzhong Li et al., metformin may protect against cisplatin‐induced renal cell apoptosis by activating AMPKα.^30^ Aside from limited advancement in our understanding of the mechanisms that underlie apoptosis in AKI, we know very little about the role of tubular apoptosis in rhabdomyolysis‐induced AKI.^18^ In the current study, we initially demonstrated that PKC‐δ was induced by glycerin and myoglobin in renal tubular cells and C57BL/6 mice, respectively. Interestingly, the inactivation of PKC‐δ by rottlerin markedly reduced glycerine‐induced death in C57BL/6 mice and also attenuated the progression of glycerine‐induced AKI. Mechanistically, we found that the PKC‐δ/p38MAPK and ERK1/2 signalling pathways mediated myoglobin‐induced renal cell apoptosis and the expression of TNF‐α and IL1‐β. These data suggested that the inhibition of PKC‐δ has a beneficial effect on AKI induced by rhabdomyolysis.

The function of PKC‐δ in apoptosis depends on cell types and stimulating factors.^26^, ^31^, ^32^, ^33^, ^34^ In tumour cells, PKC‐δ usually antagonizes apoptosis.^16^ In a previous study, we revealed that PKC‐δ induced renal cell apoptosis and then led to the progression of nephrotoxicity induced AKI.^17^, ^18^ A more recent study reported that PKC‐δ promoted the apoptosis in mitochondria and caused AKI induced by cold storage–associated kidney transplantation.^19^ Consistently, we also found that PKC‐δ induced apoptosis in renal tubular cells to promote the progression of rhabdomyolysis‐induced AKI. This effect was supported by two lines of evidence. First, rottlerin, a PKC‐δ pharmacological inhibitor, attenuated the rhabdomyolysis‐induced AKI accompanied by a reduction of renal cells (Figure 2). Secondly, rottlerin also ameliorated myoglobin‐induced renal cell apoptosis (Figure 4). Collectively, these data confirmed that PKC‐δ plays an apoptotic role during rhabdomyolysis‐induced AKI.

Recent studies suggested that rottlerin non‐selectively inhibits PKC‐δ and other enzymes, including PKC‐α and PKC‐η.^35^, ^36^ Therefore, this inhibition experiment alone is insufficient to illustrate the specific function of PKC‐δ. To further avoid the non‐specific effects of rottlerin, we used PKC‐δ‐KD and PKC‐δ‐CF to suppress or overexpress PKC‐δ, respectively. Previous studies reported that the P38MAPK and ERK1/2 pathways are related to apoptosis and inflammation.^37^, ^38^, ^39^ The p38 MAPK (mitogen‐activated protein kinase) signalling pathway conducts diverse cell functions by aiding cells to process various signals and is known to participate in cell apoptosis induced by different stimuli.^40^ The ERK1/2 pathway is associated with the inflammatory responses of irritated cells.^41^ In the present study, we demonstrated that PKC‐δ positively regulated the P38MAPK and ERK1/2 pathway to mediate apoptosis and inflammation. This was supported by specific evidence. First, PKC‐δ‐KD attenuated myoglobin‐induced apoptosis in renal cells and the expression of TNF‐α and IL1‐β by suppressing the p38MAPK and ERK1/2 signalling pathways (Figure 5). Secondly, PKC‐δ‐CF enhanced them by activating the p38MAPK and ERK1/2 signalling pathways (Figure 6). These results suggested that the PKC‐δ/p38MAPK and ERK1/2 axis mediated rhabdomyolysis‐induced AKI. Still, there are certain deficits in this research. Firstly, we used only inhibitors and plasmids to alter the expression of activated PKC‐δ, while more concrete evidence would be presented should specific genomic knockout of PKC‐δ in renal tubules was applied. Secondly, we did not investigate the functions of PKCα and PKC‐η even though they were also activated in glycerin‐induced AKI. What's more, how glycerin/MYO caused PKC‐δ activation is yet to be discovered. Further exploration is required to extend the findings in this study.

**FIGURE 7:**
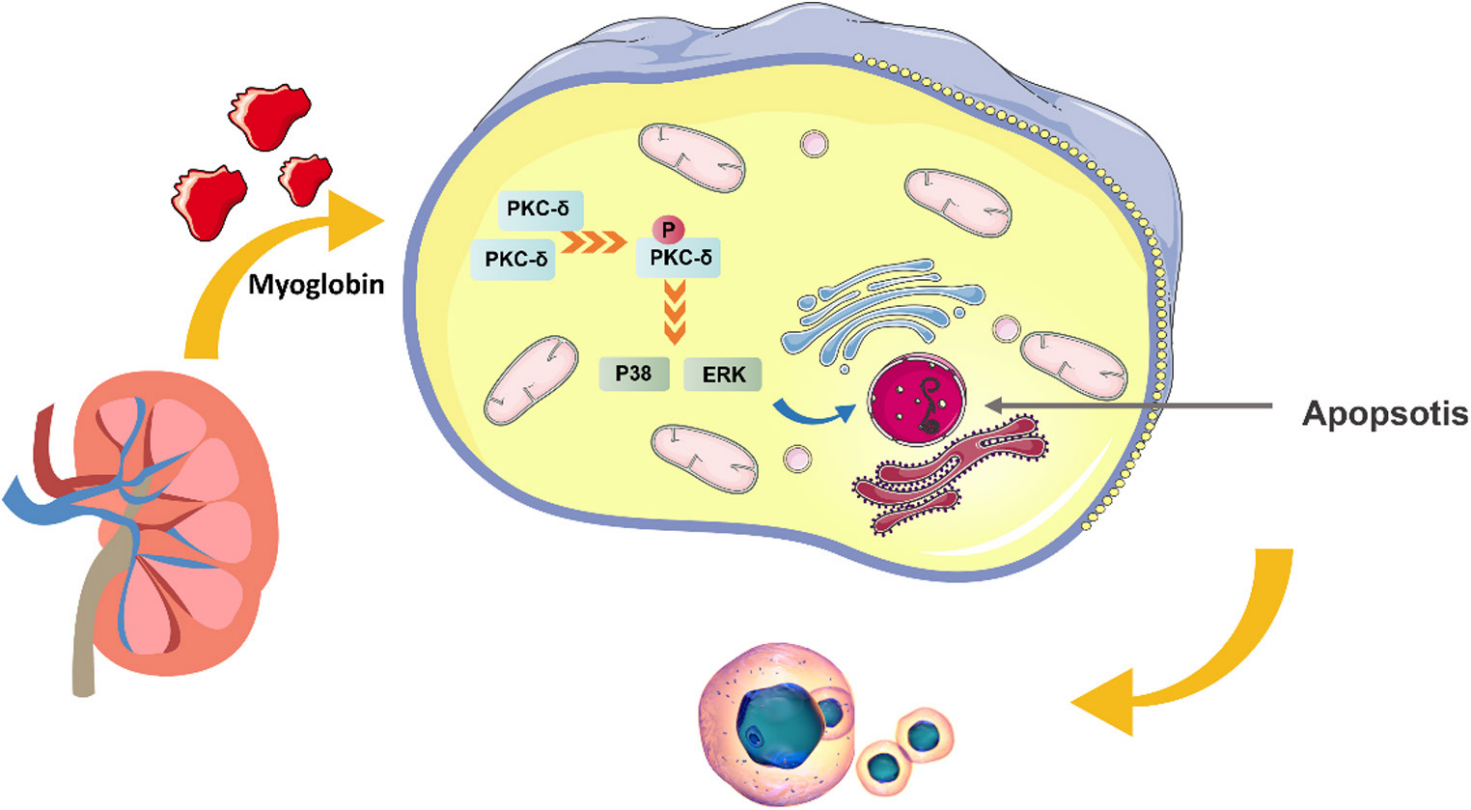
Mechanism of inhibiting PKC‐δ to reduce rhabdomyolysis‐induced AKI. Myoglobin can activate PKC‐δ and promote cell apoptosis through p38 and ERK signalling pathways

In conclusion, our research revealed that PKC‐δ mediates rhabdomyolysis‐induced AKI *via* the activation of the P38MAPK and ERK1/2 pathways (Figure 7), thus providing evidence that PKC‐δ is a potent therapeutic target for rhabdomyolysis‐induced AKI.

## CONFLICT OF INTEREST

The authors have no conflict of interest.

## AUTHOR CONTRIBUTION

**Dengke wu:** Data curation (lead); Investigation (lead); Validation (lead); Writing – review & editing (equal). **Jian Pan:** Formal analysis (equal); Methodology (equal); Resources (equal); Visualization (equal); Writing – review & editing (equal). **Dongshan Zhang:** Conceptualization (lead); Funding acquisition (equal); Writing – original draft (lead); Writing – review & editing (equal).

## Supporting information

Fig S1‐S5

## Data Availability

The data used and/or analyzed are available from the corresponding author by reasonable request.
