# Supplementary material for: Inhibition of PKC‐δ reduce rhabdomyolysis‐induced acute kidney injury
Source: J Cell Mol Med. 2022 May 2;26(11):3243–53. doi: 10.1111/jcmm.17331 (PMC9170808; doi:10.1111/jcmm.17331)
Supplement: Supplementary file 1 — Fig S1‐S5 [file JCMM-26-3243-s001.docx]

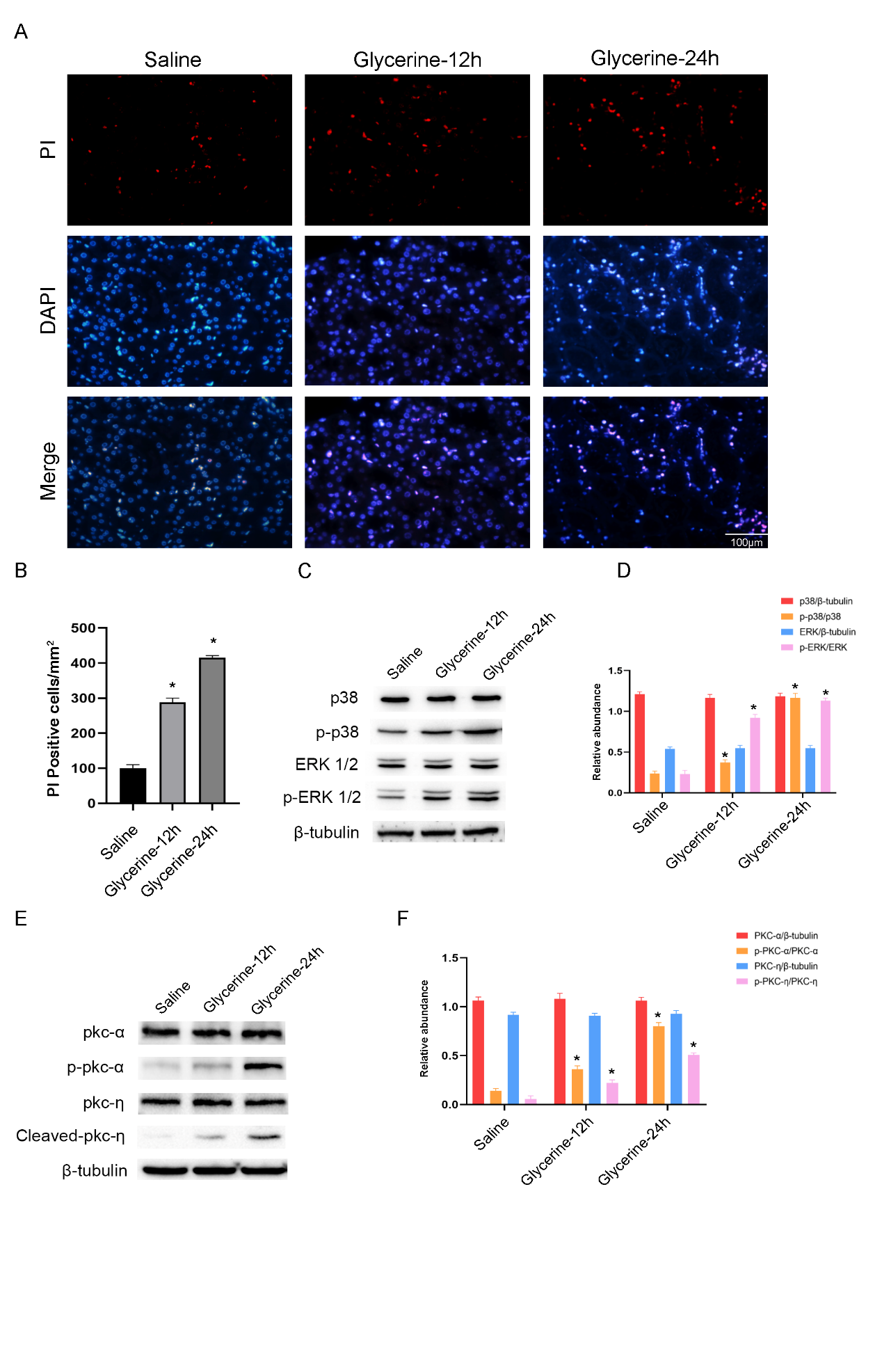


**Figure S1：Glycerin induced renal cell apoptosis and expression of p-p38, p-ERK1/2 and p-PKCs in mice kidney.** WT mice were injected intramuscularly with saline or 50% glycerine(8ml/kg) for 12 or 24 hours. (A)Representative images of PI staining. (B) Quantitative analysis of PI positive cells. (C) Representative immunoblots of p-p38 and p-ERK1/2 activation in whole kidney lysate. (D) Grayscale image analysis between them. (E) Representative immunoblots of PKC-α and PKC η activation in whole kidney lysate. (F) Grayscale image analysis between them. Original magnification, x200. Scale bar, 100μM. Data are expressed as mean ± SD (n=6). * P<0.05 versus Saline group.


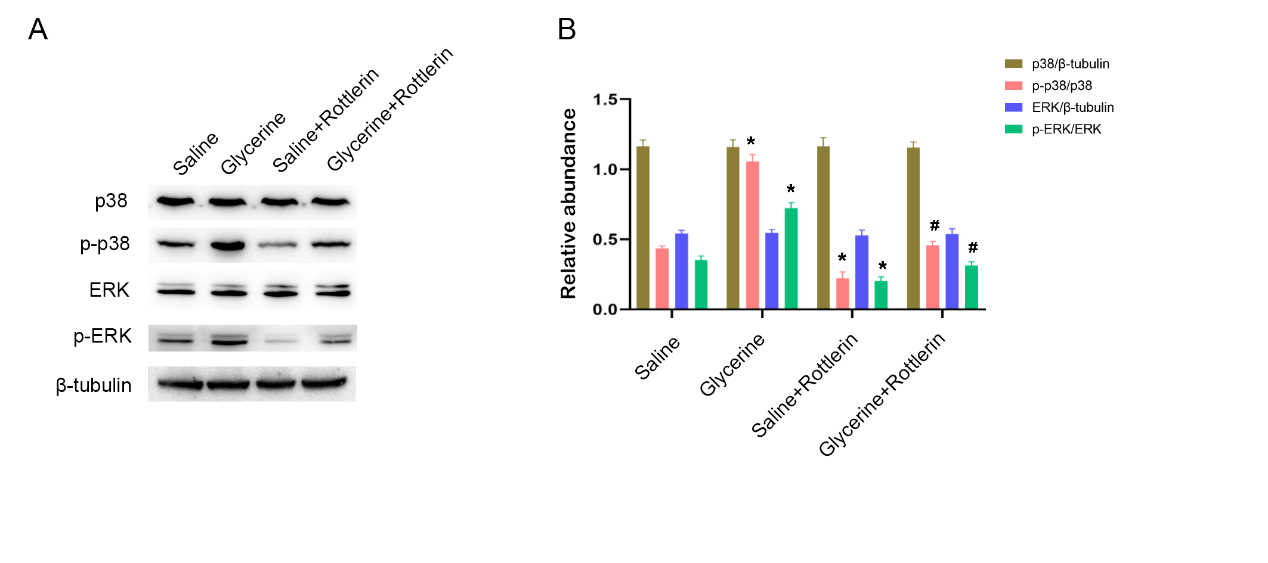


**Figure S2**：**Rottlerin mitigated** **glycerin induced activation of p38 and ERK1/2 in mice kidney.** (A) Representative immunoblots of p-p38 and p-ERK1/2 activation in whole kidney lysate. (B) Grayscale image analysis between them.* P<0.05 versus Saline group. #P<0.05 versus glycerin group.


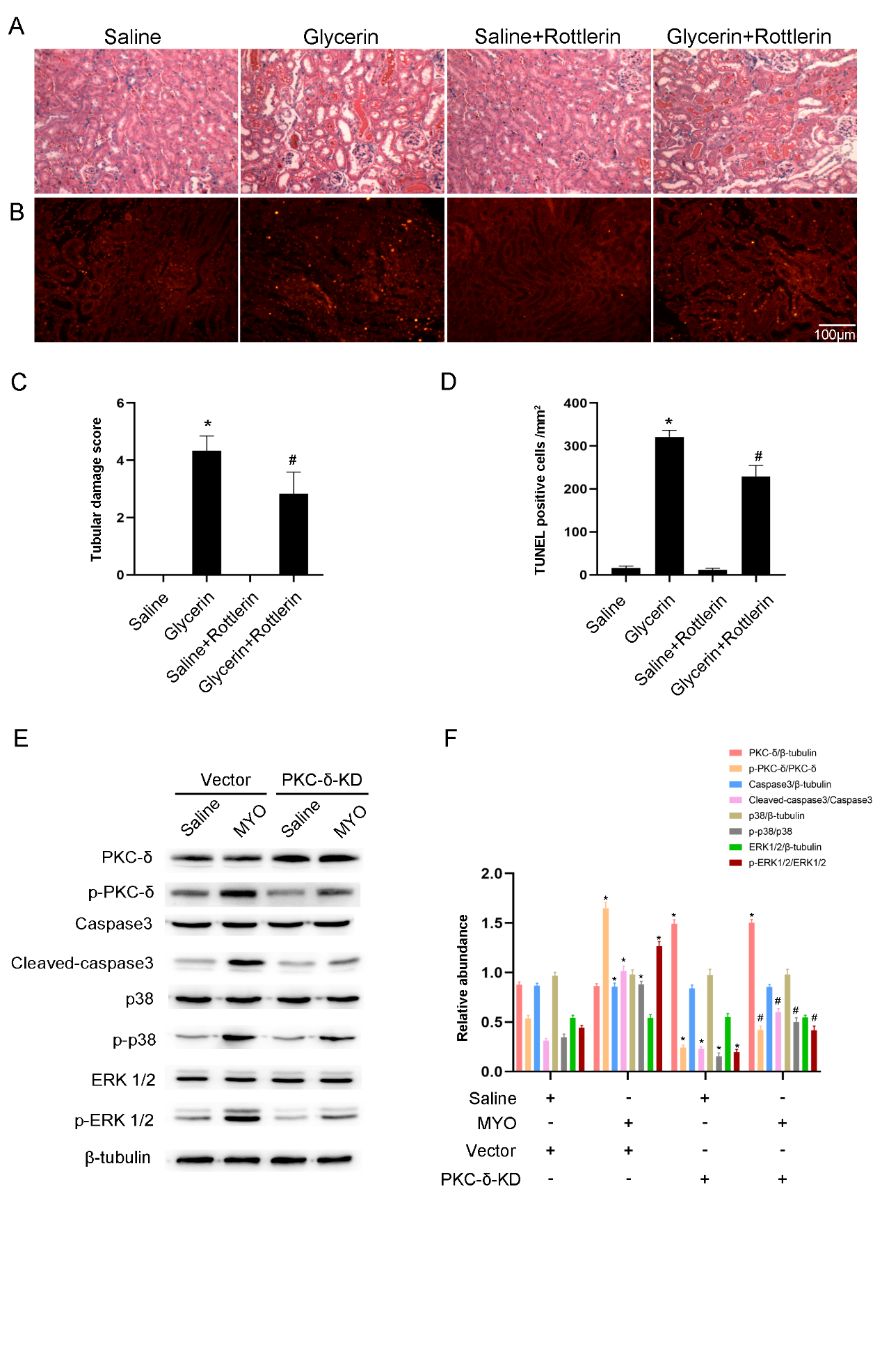


**Figure S3：****Rottlerin and** **PKCδ plasmid attenuated renal damage caused by Glycerin and MYO in mice.** Mice were treated with saline or glycerin with or without rottlerin, or treated with saline or MYO with vector or PKCδ plasmid transfection. (A&B) Representative images of H&E staining and TUNEL staining. (C&D) Quantitative analysis of tubular damage and TUNEL positive cells. (E)Immunoblots of PKC-δ，p38 and ERK1/2 activation in whole kidney lysate. (F) Grayscale image analysis between them. Original magnification, x200. Scale bar, 100μM. Data are expressed as mean ± SD (n=6). * P<0.05 versus Saline group. ^#^P<0.05 versus glycerin group.


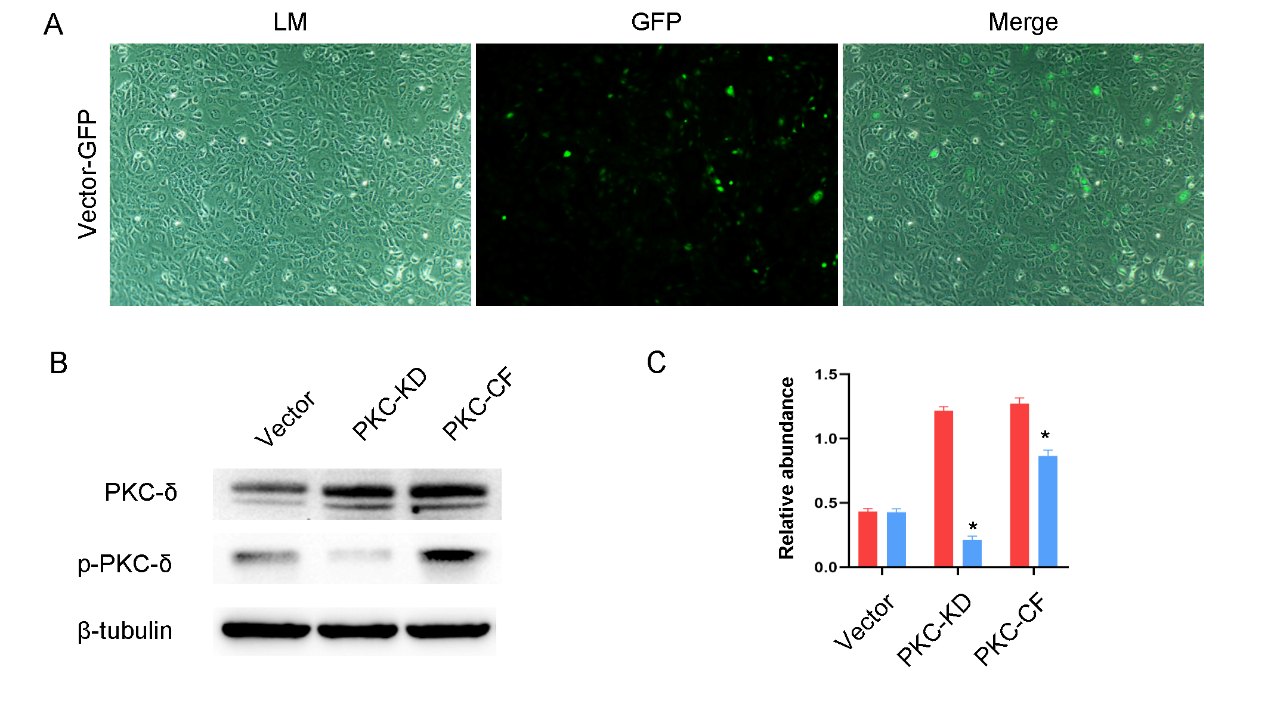


**Figure S4：Detection of PKC-δ expression in BUMPT cells after transfection of** **vector, PKC-KD and PKC-CF plasmid.** BUMPT cells were transfected with vector, PKC-KD and PKC-CF plasmid then cultured for 24h before collection. (A) Immunofluorescence detection after GFP+ vector transfection. (B) Immunoblots of PKC-δ activation in whole kidney lysate. (C) Grayscale image analysis between them. Original magnification, x200. Scale bar, 100μM. Data are expressed as mean ± SD (n=6). * P<0.05 versus vector group.


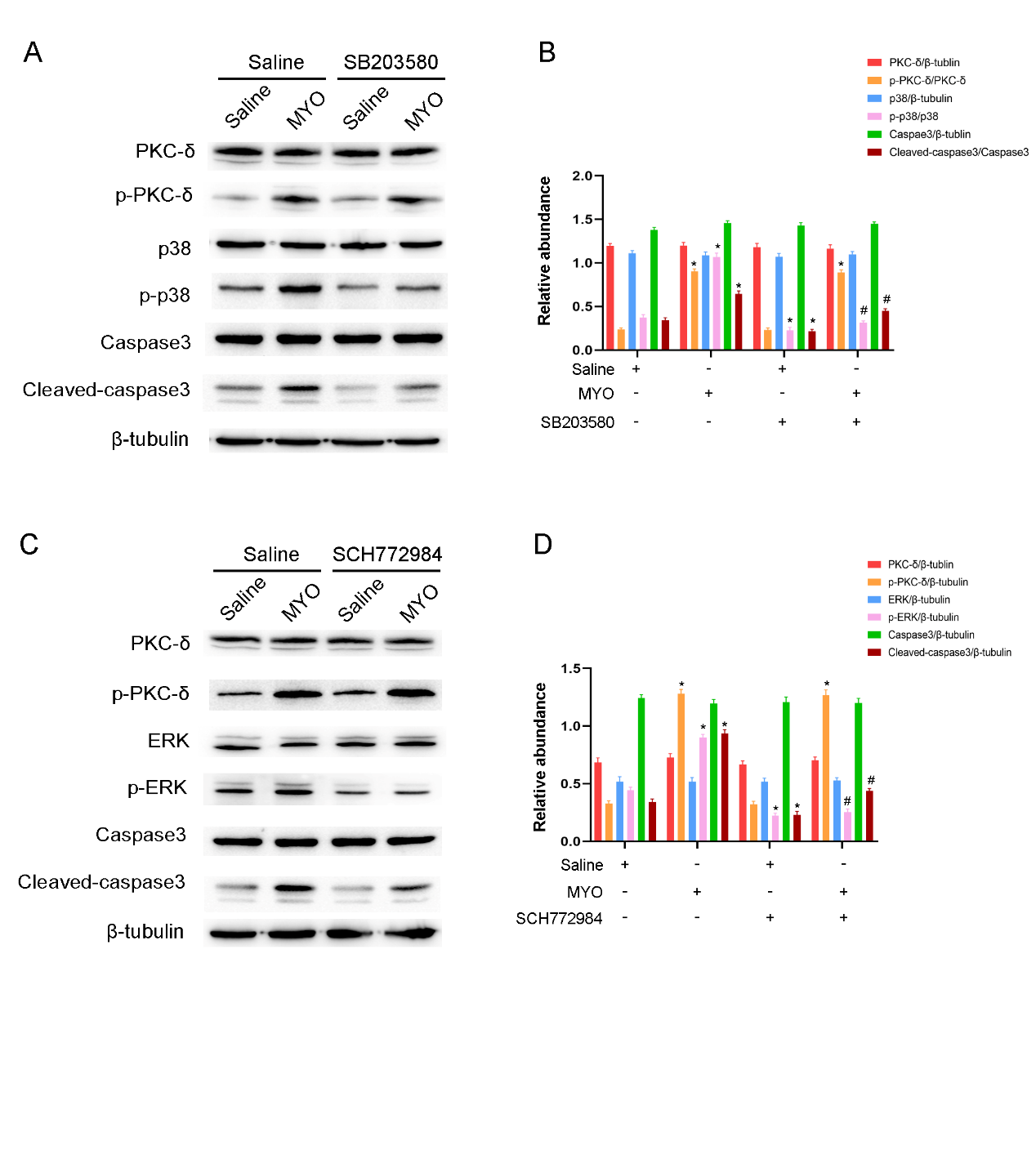


**Figure S5: Inhibitor of p38 MAPK and ERK1/2 both alleviated cell apoptosis induced by myoglobin.** BUMPT cells were treated with saline or myoglobin(10mg/ml), with or without p38 MAPK inhibitor (SB203580) or ERK1/2 inhibitor (SCH772984). (A)Representative immunoblots of PKCδphosphorylation, Caspase3 activation, p38 MAPK phosphorylation and ERK1/2 phosphorylation in cell lysate. (B, C&D) Grayscale image analysis between them. Data are expressed as mean ± SD (n=6). *P<0.05 versus Saline group. ^#^ P<0.05 versus MYO treated group.
